# Complex regional pain syndrome what is the outcome? ‐ a systematic review of the course and impact of CRPS at 12 months from symptom onset and beyond

**DOI:** 10.1002/ejp.1953

**Published:** 2022-05-04

**Authors:** Selina Johnson, Fiona Cowell, Sharon Gillespie, Andreas Goebel

**Affiliations:** ^1^ Walton Centre NHS Foundation Trust Liverpool UK; ^2^ Pain Research Institute Faculty of health and life Sciences University of Liverpool Liverpool UK; ^3^ Liverpool University Hospitals Foundation Trust (LUHFT) Liverpool UK

## Abstract

**Background and Objective:**

To improve CRPS treatment, it is imperative to understand the nature, degree and relative importance of ongoing problems associated with CRPS. The objective of this systematic review was to summarize the published data concerning measures of function and impact including occupational parameters, of CRPS at 12 months from symptom onset and beyond.

**Databases and Data Treatment:**

MEDLINE, EmBase and PsychINFO were searched (inception to May 2021). Study cohorts were eligible if they included; adult patients with the primary complaint of CRPS ≥12 months duration, outcomes that reported change in CRPS signs and symptoms, and physical and social function. Prospero registration: CRD42021241785.

**Results:**

Twenty‐two included studies suggest that pain and motor dysfunction are the most dominant long‐term features of CRPS, persisting for 51%–89% of patients at ≥12 months. On average for all patients who had CRPS at baseline, grip strength was found to be reduced by 25%–66%, and range of motion reduced by 20%–25% at ≥12 months. Such losses were associated with physical and social disability. Thirty to forty percent of all patients did not return to work and a further 27%–35% of persons returned to work but required some form of workplace adaptation, although the quality of this data was poor. Quality assessment highlighted limitations in the literature, such as high attrition bias and variations in diagnostic criteria.

**Conclusions:**

Results provide first‐time quantitative data including specific evidence about losses to motor function and long‐term compromises to work status. Results demonstrate that the ongoing impact of one episode of CRPS on limb function and work status is relatively high.

**Significance:**

This review provides first‐time clarity in relation to outcomes of limb function and work status associated with an episode of CRPS, beyond 12 months from onset. Results demonstrate that the long‐term impact of an episode of CRPS on these outcomes is much larger than previously described, and thus also illustrates how the wider health economic impact of CRPS is not yet fully understood. We additionally highlight the need for future research that identifies long‐term predictors, and treatments that can foster good functional and occupational recovery.

## 1 INTRODUCTION

Complex regional pain syndrome (CRPS) is a painful and debilitating disorder usually occurring after limb injury (Goebel et al., 2018, Gougeon et al., 1982). Typical symptoms include limb‐restricted painful skin sensitivity, swelling, colour changes, temperature changes, problems with initiation and control of movement, and feelings of disconnection with the affected limb (Alam et al., 2019; De Boer et al., 2011). Diagnosis of CRPS is confirmed by the presence of these characteristics (Bruehl et al., 1999; Harden et al., 2010).

People with CRPS frequently struggle to use their affected limb and maintain functional, occupational and social activities. The health‐related quality‐of‐life (QOL) in persistent CRPS is low in comparison with other long‐term conditions such as diabetes and chronic lung disease (van Velzen et al., 2014).

CRPS is typically monophasic, but there is considerable variation in the initial severity of‐ and later recovery from CRPS (Kemler and Furnée, 2002). For example, an early prospective study of 27 CRPS patients receiving no treatment for their condition demonstrated that only 1 patient continued with signs and symptoms of CRPS at 1‐year (Zyluk, 1998); while other studies using more detailed outcome measures have reported that even patients who substantially improve often have ongoing significant long‐term problems affecting their QOL (Bean et al., 2014b; De Mos et al., 2009). What is measured and what is considered significant in terms of CRPS outcome is inconsistent (Llewellyn et al., 2018). The relative individual impact of CRPS in the longer term is therefore poorly understood, which makes it difficult for clinicians and researchers to fully appreciate the true health‐economic impact associated with CRPS. To improve CRPS treatment and justify when and how health care resources could be best utilized, it is therefore imperative to better understand the nature, degree, and relative importance of any ongoing problems.

An earlier systematic review comprehensively discussed CRPS symptom recovery in 2012 (review®) (Bean et al., 2014b). The authors concluded that although many CRPS patients demonstrate good improvements within 6 to 13 months, a significant number, even within that improving group experience lasting symptoms. The primary focus of this 2012 review was on symptom recovery. The review highlighted that data quality was poor, and outcome measurements were variable and often limited suggesting that more research was needed. Information relating to how CRPS specifically impacts disability and occupational status was limited given the focus of the review. Information regarding the functional and occupational impact of CRPS is needed to understand the long‐term individual health economic impact of CRPS, and ensure treatment supports recovery that is meaningful to patients. Therefore, there was good rationale to update the previous review to provide specific information regarding the long‐term physical and social/occupational impact of CRPS symptoms. The present review aims to summarize the published data concerning the impact of CRPS symptoms, specifically the physical and occupational impact of symptoms, at 12 months from symptom onset and beyond.

## 2 METHODS

A systematic review of the literature was conducted, focusing on the nature and extent of CRPS symptoms at 12 months and beyond. The aim of the review was to examine the literature and to summarize the published data concerning the course and impact of CRPS symptoms over time. Trial registration: Prospero CRD42021241785.

### 2.1 Search strategy

To ensure the systematic review reflected an update of review® congruent electronic databases and search terms were used. EMBASE, MEDLINE and PsycINFO and reference lists were searched, from inception until May 2021 (search date). Example search strategy (S1).

### 2.2 Selection of studies

Study eligibility was constructed using PICO components. All abstracts were reviewed using an inclusion/exclusion tool (Table S1). Studies of adults (+18 years) with the primary complaint of CRPS ≥12 months duration, or which included cohorts followed up for ≥12 months from symptom onset, were included. Studies using outdated CRPS terms of algodystrophy, sudecks and reflex sympathetic dystrophy (RSD) were also included. Studies were included if their stated aim was to investigate the outcome, course, severity and prognosis of CRPS. Studies were excluded if they; had a sample size of ≤20 (such samples were considered too small to draw yield reliable or precise estimates (Hackshaw, 2008), assessed response to treatment, or were not published in English or French. Articles were additionally excluded if they were only available in abstract format, were based on paediatric cohorts (due to suggested differences in presentation) or had follow‐up or response rates <50% (Bean et al., 2014b).

### 2.3 Quality assessment

The Quality of included studies was assessed by two authors using the Joanna Briggs Institute (JBI) critical appraisal checklist for studies reporting prevalence data (Munn et al., 2015); any discrepancies were resolved by a third. For each question, each study was scored positive (Y), negative (N), or unclear (U) (Table S2). In keeping with review®, attrition rates of >20% or response rates of <75% were considered at high risk of bias.

### 2.4 Data extraction

Included abstracts proceeded to a full‐text review. Extraction data included study type, sample and method, diagnostic criteria, duration of CRPS at baseline, duration of CRPS at follow‐up, the timing of assessments, and relevant outcome measures. Relevant outcome measures included data concerning resolution of CRPS, change in CRPS Signs and symptoms, and physical and social disability (e.g. occupational adjustments).

### 2.5 Data synthesis

Due to significant heterogeneity in research methods, it was not possible to quantitatively pool data. Therefore, data were synthesized according to study type and findings of the relevant outcome measures were reported. For outcomes describing changes in signs and symptoms the review focusses on the diagnostic categories ‘sensory/pain’ and ‘motor function’ (weakness, range of movement, quality of movement) (Harden et al., 2010); this is because signs and symptoms in these categories have been consistently described as the most persistent and disabling (Bean et al., 2014b; Llewellyn et al., 2018); in contrast we found vasomotor and sudomotor features typically exhibited greater improvements and were less disabling (Table S3). Within each section, we highlight which studies were included in the review® and where some of these earlier studies did not make the threshold for the current review. Overall results are discussed in the context of review®, with a focus on quantification of outcomes pertaining to physical and occupational function.

## 3 RESULTS

**FIGURE 1:**
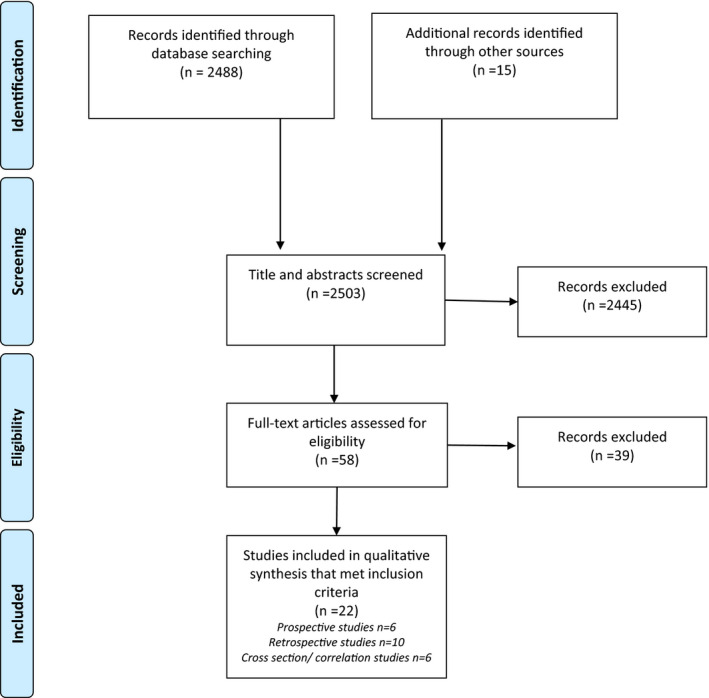
Prisma diagram flowchart of inclusion

Electronic database search yielded 2488 papers‐ 747 papers were published since review®. A further 15 papers were identified from hand searches. 58 of these 2503 were selected for a closer review and examined in detail. The second author screened any study where uncertainty remained about inclusion/exclusion criteria, and a consensus decision was made. Of the 58 studies, 22 met the inclusion criteria (Figure 1). Of these 22, 15 studies were congruent with those reviewed in review^®^ and 7 were published later.

Of note, review® had included 3 additional studies, which did not meet the thresholds for this present review (Table 1), study characteristics (Table 2).

**TABLE 1:** Studies excluded from earlier review

Reference	Study type	Reason for exclusion	Detail
1. Atkins et al. (1989)	Prospective	Follow up <12 months	Follow up 6 months
2. Fialka et al. (1991)	Retrospective	Sample size <20	Sample size 17
3. Zyluk (2001)	Retrospective	Follow up <12 months	Follow up 11 months

**TABLE 2:** Study characteristics

Reference and Year	Setting and location	Sample and method	Diagnostic criteria	Baseline Assessment	Duration of CRPS at Follow up
Prospective cohort studies					
Zyluk (1998)	Surgical dept. Poland	30 RSD patients followed over 13months all of which received no treatment during this period. 3 patients with severe symptoms withdrew for treatment, therefore symptoms in remaining 27 are reported	Own	At time of diagnosis (mean of 12 weeks post onset)	6 and 13 months
Beerthuizen et al. (2012)	Orthopaedic clinic Netherlands	596 consecutive fracture patients reviewed in respect to different diagnostic criteria at 3 time points	Harden and Bruehl, IASP Veldman	Within 2 weeks of fracture	6 weeks, 3 months and 1 year
Bickerstaff and Kanis (1994)	Casualty dept. UK	274 colles fracture patients were reviewed monthly until symptoms resolved. 77/274 = CRPS	Atkins	7 weeks (After fracture)	6 and 12 months
Laulan et al. (1997)	Orthopaedic dept France	100/125 distal radius fracture patients followed for 12 months. 26/100 = CRPS	Own	Within 1 week of fracture	12 months
Bean et al. (2015)	Community and orthopaedic hand and physio clinics, New Zealand	66 CRPS patients with CRPS diagnosis, subjective and objective measures taken at 3 time points and used in analysis of predictors of outcome	IASP	Within 12 weeks of symptom onset	6 and 12 months
Bean et al. (2016)	Community and orthopaedic hand and physio clinics, New Zealand	59 CRPS patients from Bean et al. (2016) cohort who additionally met the Budapest criteria, subjective and objective measures taken at 3 time points and used in analysis of extent of recovery	Budapest	Within 12 weeks of symptom onset	6 and 12 months
Retrospective studies					
Gougeon et al. (1982)	French Society of Rheumatology.	573 RSD cases identified from a survey of society members, 370 files selected for review, of 227 reported RSD until resolution	Not described	unclear	6, 12, and 36 months.
Bejia et al. (2005)	Rheumatology Department, Tunisia	Cohort sample of 60 algodystrophy cases seen between 1989–2003	Not described	Within 13 weeks of symptom onset	15 months
De Mos et al. (2009)	Primary care Netherlands	CRPS patients were retrospectively identified (1996 to 2005) on the Dutch general practitioner's database (IPIC), 102 patients with min duration of 2 years were assessed. 75 with matched controls completed 1 × study visit comparing signs and symptoms	IASP	1st mention on database	Mean 5.8 years (range 2.1 to 10.8)
Dumas et al. (2011)	Occupational health centres France	55 CRPS patients identified from medical records (unclear whether consecutive). Patients telephoned to administer questionnaire	Veldman	n/a	Duration of follow up unclear
Subbarao and Stillwell (1981)	Unclear clinical setting USA	125 patients identified from medical notes, 123 questionnaires sent, 77 respondents	From Perez et al. (2010)	22 weeks since onset	Mean 22 months
Galer et al. (2000)	Department of Pain Medicine USA	55 CRPS patients treated from 1997 to 1998 sent questionnaires	IASP	n/a	Mean 33 months
Geertzen et al. (1998)	Rehab dept. Netherlands	93 patients treated between 1988–1994 invited for follow up, 65 (70%) responded.	Own	n/a	Mean 5.5 years (range 3–9 years)
Sharma et al. (2009)	RSD Association of America Website, United States	A survey of 75 questions was hosted on the Web site of the Reflex Sympathetic Dystrophy Syndrome Association of America for 5 months.	Budapest	n/a	Mean 5.5 years
Savaş et al. (2008)	Department of physical medicine and rehab Turkey	30 patients discharged with a good outcome attended for physical assessment	From (1)	Unclear	18 months
Ehrler et al. (1995)	Rehabilitation centre, France.	Follow up questionnaire sent to 47 patients who had taken art in a study 9 years earlier. 25 responded (53%)	Not described	2 groups, 1 = 1 week, 2 = 28 weeks	9 years later
Cross sectional/correlation studies					
Bean et al. (2014a)	Interdisciplinary pain centre New Zealand	88 consecutive CRPS patients (Jan 2009‐Dec 2011). Assessed the relationships between psychological factors, pain, and disability in CRPS, compared with 88 low back pain patients	Budapest	n/a	CRPS 43.25 months (±53.73) LBP 43.42 (±53.14)
Antunovich et al. (2020)	Interdisciplinary pain centre New Zealand	53 CRPS completed questionnaires. Multiple regression analyses were used to determine whether illness perceptions were associated with pain intensity, disability, depression, and kinesiophobia	Budapest	n/a	62.14 months (±50.15)
De Jong et al. (2011)	Pain clinic Netherlands	Investigated pain‐related fear in CRPS in 2 cross‐sectional studies. Study I = 79 patients with early CRPS, Study II =107 patients with CRPS	Veldman	n/a	STUDY 1– 48.1%< 1 month, 35% 1–6 months, 21% >6months. STUDY 2: 1% <1 m, 99% = >6/12
Schwartzman et al. (2009)	Pain clinic USA	Retrospective review of 656 patients with CRPS of at least 1‐year duration using data extracted from a patient questionnaire administered over 10.5 years.	Budapest	n/a	1 – 46 years, average unclear.
Veldman et al. (1993)	Department of Surgery, Netherlands	Retrospective review of 829 consecutive RSD patients. Patients were divided into 4 groups based on CRPS duration and diagnostic criteria symptom prevalence described	Veldman	n/a	Group 1: 0–2 mo (*n* = 156) Group 2: 2–6 mo (*n* = 242) Group 3: 6–12 mo (*n* = 200) Group 4: >12 mo (*n* = 231)
De Boer et al. (2011)	5 × Outpatient clinics participating in the TREND knowledge consortium (Trauma Related Neuronal Dysfunction)	Replicated Veldman study but used IASP diagnostic criteria *n* = 692	IASP	n/a	Group 1: 0–2 mo (*n* = 48) Group 2: 2–6 mo (*n* = 211) Group 3: 6–12 mo (*n* = 70) Group 4: >12 mo (*n* = 352)

Abbreviations: CRPS, complex regional pain syndrome; IASP, International association for the study of pain; LBP, low back pain; n/a, not applicable; RSD, reflex sympathetic dystrophy.

### 3.1 Quality assessment

Study quality was variable (Table S3); below we have listed the main identified limitations.

1. Non‐uniform choice of diagnostic criteria. In 2010 the Budapest criteria became the recognized diagnostic gold standard (Harden et al., 2010), 2/14 of studies published before 2010 and 5/8 of studies published after 2010 used these criteria.
2. Most studies (18/22) did not include samples representative of the wider CRPS population and were conducted within a specialist setting. Additionally, sample size justification was considered a source of bias in 10/22 included studies.
3. As we wished to understand the natural course of CRPS, we therefore also considered whether samples represented ‘inception‐cohorts’. These samples are recruited within 3 months of CRPS onset (Bean et al., 2014b) and accounted for 7/22 studies. While other studies may have excluded CRPS patients who did not seek treatment or whose CRPS significantly improved prior to the point of inclusion.
4. All studies defined their outcomes; 13/22 studies used validated outcome measures; 9/22 studies included an objective measure.
5. 12/22 studies performed relevant statistical testing, for example reporting statistically significant change between groups or change in symptoms/impact over time.
6. 14/22 were considered high at risk of bias in terms of response and attrition rates and only one study described the management of missing data (Bean et al., 2014a).

### 3.2 Prospective studies (*n *= 6)

Of these, four were in review® (Bickerstaff & Kanis, 1994; Laulan et al., 1997; Zyluk, 1998); one study from review® was excluded. For this review, both baseline and >=12 months outcomes are reported (Table 3).

**TABLE 3:** Results of the prospective studies

Reference	n	Outcome timing	Outcome Domains				
			Recovery/severity	Sensory/Pain outcomes	Motor function outcomes	Physical and social disability outcomes	Work status outcomes
Beerthuizen et al. (2012)	596	T1‐6 weeks after POP removal T2‐3 months T3‐ 1 year	No. meeting diagnostic criteria. For H&B diagnostic criteria: T1= 5% (*n* = 30), T2= 11% (*n* = 66), T3 = 7% (*n* = 42)	n/a	n/a	SF36 Physical component T1: CRPS = 27.3 (7.25), control = 34.6 (8.56) non CRPS. T2: CRPS = 30.8 (8.34), Control = 44.9 (10.0) Further raw scores including for T3 are not provided	n/a
Bickerstaff and Kanis (1994)	77	T1‐3 months post # T2 6 months T3 12 months	n/a	A) % of patients with ongoing pain T1‐ 60%, T2‐ 30%, T3‐ 14% B) % of patients with ongoing tenderness‐ affected vs unaffected (dolorimeter) T1 = 77%, T2 = 55%, T3 = 18%	A) % CRPS patients with stiffness T1 = 89%, T2 = 76%, T3 = 65% B) Average grip reduction for all patients T1 = unclear, T2 = 50% with a diagnosis of CRPS, control=parity with non‐injured limb T3 = 45% with a diagnosis of CRPS	n/a	n/a
Laulan et al. (1997)	100	T1‐ Within 1 week of # T2‐12 months	No. meeting diagnostic criteria. T1 = 26% (26/100) T2 = 15% (15/26 diagnosed at T1).	% With pain T1 = 73% T2 = 19%	T2 only (*n* = 100) *Good =< 25% loss,* Grip = 64%, ROM = 39% *Moderate =>25% loss* Grip = 23% (*n* = 2 with CRPS), ROM = 38% *Poor=>50% loss* Grip = 13% (all had CRPS), ROM = 25% (*n* = 23 with CRPS)	n/a	n/a
Zyluk (1998)	30	T1 within 12 weeks of fracture T2 6 months T3 13 months	No fulfilling diagnostic criteria T1‐ all T2‐ not reported T3‐ 1/27 (3 withdrew for treatment)	% With pain T1‐all T2‐29% T3‐7%	% Of grip in comparison to unaffected T1‐0.4%, T2‐17%, T3‐ 45% T3 only (*n* = 27) responder outcome: Good=No pain and full finger flexion = 73% Moderate = pain after load and <3cm loss of finger flexion = 13% Poor = persistent pain and >3cm loss of finger flexion = 13%	n/a	n/a
Bean et al. (2016)	56	T1‐ <12 weeks of symptom onset T2 6 months T3 12 months	No. meeting diagnostic criteria. T1 = 100%, T2 = 42.1%, T3 = 26.8% Severity score (0–17) T1 = 12.18 (2.02), T2 = 8.52 (3.33), T3 = 6.75 (4.07)	NRS T1 = 4.22(2.22) T2 = 2.35 (2.10) T3 = 1.77 (2.06)	% of range of movement regained (average of all patients). T1 = 57.9% (28.94) T2 = 81.75% (18.82) T3 = 79.82% (22.54)	Disability index score T1 = 38.12 (14.19) T2 = 19.19 (15.47) T3 = 15.03 (16.43)	Working patients represented 69.5% of the total sample. Figures below indicate percentages who returned to work with the 69.5% of working patients taken as 100%. T1 = 42.9%, T2 = 67.5% T3 = 64.1% Work hours: T1 = 16.46 (20.48), T2 = 25.64 (21.25), T3 = 25.97 (23.44). Normal fulltime hours not indicated
Bean et al. (2015)	66	T1‐ <12 weeks of symptom onset T2 6 months T3 12 months	Severity score (0–17) T1 = 12.64 (2.41), T2 = 8.75 (3.26), T3 = 6.80 (4.07)	A) % With pain T1 = 100%, T2 = 88%, T3 = 73% B) NRS T1 = 5.49 (2.03), T2 = 3.43 (2.55) T3 = 2.76 (2.89) C) % allodynia T1 sub = 67%, T2 = 34%, T3 = 25% T1 obj = 43%, T2 = 19%, T3 = 18%	n/a	Disability index score T1 = 37.15 (14.33) T2 = 18.15 (15.13) T3 = 14.73 (16.07)	n/a

Note

Scores in brackets denote standard deviation, all scores represent average scores for study population of stated measure.

Abbreviations: %, percentage of total sample; #, fracture; CRPS, complex regional pain syndrome; H&B, harden and Bruehl; n/a, no outcomes for this domain recorded; NRS, numerical rating scale; POP, plaster of Paris; T1, baseline; T2, first follow up assessment; T3, second follow up assessmen.

Three studies monitored cohorts of patients following fracture over time and reported outcomes for patients with and without CRPS (Beerthuizen et al., 2012; Bickerstaff & Kanis, 1994; Laulan et al., 1997). One study followed CRPS patients who had no treatment for their condition (Zyluk, 1998).

A further two more recent prospective studies recruited patients with CRPS <12 weeks, from community and orthopaedic‐based hand and physiotherapy clinics (Bean et al., 2015, 2016). The 2015 study examined psychological predictors of outcome for patients fulfilling the IASP diagnostic criteria (*n* = 66) (Bean et al., 2015). The 2016 study included the same cohort who also met the Budapest criteria to examine the extent of recovery (Bean et al., 2016).

#### CRPS resolution

Four studies recorded CRPS diagnosis at baseline and follow up (FU) (Bean et al., 2016, Beerthuizen et al., 2012, Laulan et al., 1997, Veldman et al., 1993). One study followed a cohort that received no treatment and reported that only one patient continued to be diagnosed at 13 months (Zyluk, 1998). However, lower recovery rates were reported by other studies. At 12 months; two studies reported that 57%–63% of patients continued with CRPS post‐fracture (Laulan et al., 1997, Beerthuizen et al., 2012), and a further study reported 25% of patients continued with CRPS (Bean et al., 2016). These results indicated that symptoms of CRPS do improve for many over time, to the extent they no longer fulfil a CRPS diagnosis. Despite improvements all studies noted that few patients were ever without any CRPS symptoms at FU, with 5.4% representing the highest reported percentage of patients reporting no ongoing CRPS symptoms across studies (Bean et al., 2016).

#### Sensory/pain outcomes

In 3 studies that included a non‐CRPS control, 2 reported that patients who developed CRPS had higher pain intensity at baseline than those without (Beerthuizen et al., 2012; Laulan et al., 1997). Painful symptoms, including allodynia, tenderness and pain intensity, showed an improvement by 12 months in all studies (Table 3).

#### Motor function

Motor symptoms were measured in four studies, each using different outcome measures (Bean et al., 2016; Bickerstaff & Kanis, 1994; Laulan et al., 1997; Zyluk, 1998).

Bickerstaff and Kanis, followed 274 patients following wrist fracture of which 77 developed CRPS. 65% of CRPS patients reported ongoing stiffness at 12 months, compared to 20% in the control population. Grip strength was measured as a ratio of the affected hand compared to the unaffected hand. Grip strength in the *control* population had almost reached parity at 6 months but was reduced by 50% for CRPS patients, and 45% of CRPS patients at 12 months (raw data relating to SEM is not provided).

Laulan et al and Zyluk divided patients into subgroups dependent on recovery at 12 and 13 months respectively. Laulan et al objectively measured wrist mobility and grip strength (dynamometer) and compared measures with the uninvolved contralateral side (*n* = 100). A reduction of <25% in wrist mobility or grip strength was described as a good outcome, *moderate* outcome >25% loss, and poor outcome >50% loss. At 1 year for mobility 39% achieved a good outcome, 38% moderate and 23% poor (15 had CRPS). Grip strength: 64% good, 23% moderate (2 still had CRPS) and 13% poor (all still had CRPS). Zyluk (*n* = 27) grouped CRPS patients as: with good outcome (no pain and full finger flexion) =73%; moderate (pain after loading and <3 cm loss of finger flexion) =13%; and poor (persistent pain and loss of finger flexion >3cm) =13%. They additionally reported that mean grip strength (dynamometer) for the whole group increased from 0.4% of the uninvolved contralateral side at baseline, to 17% at 6 months, and 45% at 13 months.

Therefore, all three studies described that despite improvements an episode of CRPS was associated with on average losses of between ¼ ‐ ½ of grip strength at ≥12 months.

Bean et al measured wrist and ankle range of movement for patients with CRPS (*n* = 59) (Bean et al., 2016); on 12‐month objective assessment on average patients had regained 79.82% (standard deviation (SD) = 22.54) of movement compared to unaffected side and therefore had lost on average 20% of movement, and 75% reported problems with limb movement.

#### Physical and social disability

Several prospective studies used standardized measures of disability that indicated ongoing difficulty for some and improvements for others. Both Bean et al papers used the disability index score (Bean et al., 2015, 2016) and illustrated improvement from moderate/severe in average scores at baseline, to mild/moderate at 12 months (combined values of 37.15 to 14.74 (*p *< 0.001), and 38.12 to 15.03 (*p *< 0.001) (Bean et al., 2016).

Beerthuizen et al reported significantly lower physical component scores using the SF‐36 (*p *<0.001) at one year, for patients who fulfilled a CRPS diagnosis at baseline than those who did not (27.3 (SD 7.42) vs. 34.6 (SD SD 8.34) vs. 44.9 (SD 10.0)) (Beerthuizen et al., 2012).

The 2015 Bean et al study considered what baseline variables were predictors of recovery (Bean et al., 2015). Mixed‐effects models were conducted to identify variables associated with pain scores and disability over the 12 months. For pain, the effects of disability and anxiety on pain scores were found to be statistically significant; those with lower disability and anxiety scores at baseline had lower pain intensity over the following 12 months (*p *< 0.01 and *p *< 0.05 respectively). For disability, those with lower pain and pain‐related fear scores at baseline were less disabled over the following 12 months (*p *< 0.01 and *p *< 0.05 respectively). They suggest that pain intensity, anxiety and pain‐related fear, are associated with poorer outcomes in CRPS.

#### Work status

Only one study measured this reporting that 69.5% of the included 59 patients were working prior to CRPS onset (Bean et al., 2015). Of these 67.5% had returned to work (RTW) at 6 months and 64.1% by 12 month and worked 25.97 h (SD 23.44) per week. Pre CRPS working hours were not reported.

### 3.3 Retrospective studies (*n* = 10)

Overall, ten suitable retrospective studies were identified (Bejia et al., 2005, De Mos et al., 2009, Dumas et al., 2011, Ehrler et al., 1995, Galer et al., 2000, Geertzen et al., 1998, Gougeon et al., 1982, Savaş et al., 2008, Sharma et al., 2009, Subbarao & Stillwell, 1981) (Table 2), all of which had been included in review®, two identified from review® were excluded (Fialka et al., 1991; Zyluk, 2001) (Table 1).

De Mos et al in a high‐quality study identified patients from the Netherlands integrated primary care information project to provide a population‐representative CRPS sample (De Mos et al., 2009). The study compared outcomes in these patients with matched reference patients with identical past injuries but no CRPS development.

The remaining nine studies scored lower in terms of quality. Eight studies identified patients from patient treatment records (Bejia et al., 2005, Dumas et al 2009, Ehrler et al., 1995, Galer et al., 2000, Geertzen et al., 1998, Gougeon et al., 1982, Savaş et al., 2008, Subbarao & Stillwell, 1981), while one used an online survey hosted on a CRPS website (Sharma et al., 2009), (Table 4).

**TABLE 4:** Retrospective study outcomes reported

Reference	n	Outcome timing *(Average duration since symptom onset)*	Outcome Domains				
			Recovery/severity	Sensory/Pain outcomes	Motor function outcomes	Physical and social disability outcomes	Work status outcomes
Gougeon et al. (1982)	227	Followed up until ‘cured’ – max follow up =3 years	% cured = 78% @ 3 years *n* = 177	n/a	n/a	n/a	n/a
Bejia et al. (2005)	60	15 months	Grouped according to outcome: Very good result 16% (*n* = 10) Good result 46.5% (*n* = 28) mod result 28.7% (*n* = 17) Poor result 8.8% (*n* = 5) *(Criteria of French society of rheumatologists)*	n/a	n/a	n/a	n/a
De Mos et al. (2009)	102*	5.8 years	1. % who met IASP criteria for CRPS 64% (*n* = 65) 2. Cluster analysis to identify subgroups (*n* = 102): Best outcome 61% (*n* = 62) Moderate outcome 25% (*n* = 26) Poor outcome 14% (*n* = 14) 3. Self‐reported recovery within groups	(1)% with pain = 32% (2) Sensory score (0–2)	(1) Motor score (0–3) (2) Reduced ROM* 60% (sub), 44% (Obj) (3) Weakness* = 59% (sub), 41% (obj)	n/a	53% working at baseline (*n* = 54/102) 41% = able to previous role 28% = adapted RTW 31% = unable to RTW
Dumas et al. (2011)	55	12 months	n/a	% with pain = 60%	n/a	n/a	Able to RTW = 67% Unable to RTW = 33%
Subbarao and Stillwell (1981)	125	22 months	n/a	% with pain = 41%	Reported stiffness = 51%	Able to return to full activity = 23% 14% = considerable activity modification	61% of patients working at baseline (*n* = 77/125) 30% = RTW 31% = Retired or unable RTW 35% = Officially disabled 4% not accounted for
Galer et al. (2000)	55	33 months	n/a	Change over time Improved = 29% Unchanged = 42% Worse = 29%	Reported weakness = 52%	BPI interference score >5/10 = 75%	n/a
Geertzen et al. (1998)	99	5.5 years	RSD severity score (0–64) Unaffected side = 0.7 (1.5) affected side = 5.6 (8.6)	% with pain = 58%	Reported reduction in muscle strength = 58% Grip strength (Newtons) Affected = 170 (104) Unaffected = 231 (105)	n/a	n/a
Sharma et al. (2009)	888	5.5 years	n/a	% with pain = 79% NRS 6.9	ADLS Interference = 96% Motor worsening = 11.8%	Mobility problems = 86% Self‐care problems = 57%	Work interference = 62% (*n* = 551) Social security credit = 2/3
Savaş et al. (2008)	30	18 months	10% symptom free (*n* = 3) 90% symptomatic (*n* = 27)	Hand pain after use = 86% Hand pain at rest = 76% VAS = 2.8 (2.00)	Grip strength (kg) CRPS right = 20.32 (9.52) Control right = 30.50 (9.90) CRPS left = 16.20 (8.24) Control left = 27.86 (9.89)	DASH (0 = 100) CRPS = 55.27 ± 21.08, Control = 26.16 ± 5.56	n/a
Ehrler et al. (1995)	25	9 years	40% not normalized (*n* = 10)	% with pain = 36%	Reported reduction in muscle strength =36%	n/a	n/a

Note

All scores represent average scores for study population of stated measure.

Abbreviations: %, percentage of total sample; ADLS, activities of daily life; CRPS, complex regional pain syndrome; DASH, Disability of the arm shoulder and hand (DASH) questionnaire; figures in brackets, standard deviation; n, number; n/a, no outcomes for this domain recorded; NRS, numerical rating scale; obj, objective measure; PI, pain intensity; ROM, range of movement; RTW, return to work; sub, subjective measure.

* 75 completed physical examination as part of motor function outcomes.

#### 3.3.1 CRPS resolution

Six/ten studies reported on general recovery in respect to CRPS (Bejia et al., 2005, De Mos et al., 2009, Ehrler et al., 1995, Geertzen et al., 1998, Gougeon et al., 1982, Savaş et al., 2008). Findings were highly heterogeneous between studies with ratings ranging between 22%–90% for the number of patients who had ongoing CRPS symptoms at longer term FU.

Noting that within their respective studies patient outcomes were highly variable, two studies retrospectively grouped CRPS patients into outcome categories (Bejia et al., 2005; De Mos et al., 2009). Both studies report just over 60% of CRPS patients recovered well by 12 months, while 25%–29% achieved a moderate outcome and 9%–14% were categorized as having poor outcomes (Table 2).

#### 3.3.2 Sensory/pain outcomes

The prevalence of pain as an ongoing symptom was generally higher than reported for prospective studies. Many studies used questionnaires to collect outcomes, however, administration of these varied across all studies preventing like for like comparisons. Seven studies reported percentages of patients with ongoing pain at final FU ranging from 32%–86% (De Mos et al., 2009, Dumas et al., 2011, Ehrler et al., 1995, Geertzen et al., 1998, Savaş et al., 2008, Sharma et al., 2009, Subbarao & Stillwell, 1981). The De Mos et al study recorded that 32% of people with CRPS reported ongoing pain in the general population, which was lower than the percentages reported by the studies (De Mos et al., 2009). Unlike the other studies this included patients who may have not required or sought treatment. Within this study higher pain and high incidence of sensory symptoms at baseline were associated with poorer outcome at FU (De Mos et al., 2009).

#### 3.3.3 Motor function

Seven/ten retrospective studies report a high prevalence of problems affecting motor function, that is, weakness and stiffness; however, there was substantial variation in how this was measured (De Mos et al., 2009, Galer et al., 2000, Geertzen et al., 1998, Savaş et al., 2008, Sharma et al., 2009, Subbarao & Stillwell, 1981).

Five of these seven studies used a range of subjective measures, including (1) ‘reduced range of movement’ (60% at 5.8 years) (De Mos et al., 2009), (2) ‘weakness’ (59% at 5.8 years) (De Mos et al., 2009), (52% at 33 months) (Galer et al., 2000), (3) ‘stiffness’ (51% at 22 months) (Subbarao & Stillwell, 1981), (4) ‘reduced muscle strength’ (58% at 5.5 years) (Geertzen et al., 1998), (36% at 9 years) (Ehrler et al., 1995), and (5) ‘motor problems interfering with activities of daily life’ (96% at 5.5 years)(Sharma et al., 2009).

Three of these seven also included objective functional measures. Geertzen et al and Savas et al reported that patients had regained on average 74% of their contralateral grip strength (*n* = 65 at 5.5 years) (Geertzen et al., 1998), and 58%–66% (right versus left) of hand grip compared to matched controls respectively (*n* = 30 at 18 months) (Savaş et al., 2008). De Mos et al invited patients for a physical examination (75/102 total sample) and observed ‘weakness’ for 41% and ‘reduced range of motion’ for 44% (5.8 years) (De Mos et al., 2009). The measurement method is unclear, however, observed percentages were lower in comparison to subjective percentages reported by patients for the same features. De Mos et al report motor problems at baseline, were three times more prevalent in those with poor long‐term outcomes, compared with patients considered to have a good outcome.

#### 3.3.4 Physical and social disability

Several studies quantified disruption to activity associated with CRPS. At 22 months, Subbareo & Stillwell describe that 23% of patients returned to full activity, while 14% required considerable activity modification (Subbarao & Stillwell, 1981). Sharma et al (online survey) described mobility problems in 86% and self‐care problems in 57% (Sharma et al., 2009). Galer et al reported substantial interference in 75% of patients (score ≥5⁄10) with general activity, mood, normal work and recreational activities using the brief pain inventory interference subscale (Galer et al., 2000). Savas et al found patients had mild‐ moderate disability using the disability of the arm, shoulder and hand (DASH) questionnaire (mean score=55.27 ± 21.08 in CRPS patients and mean score=26.16 ± 5.56 in controls) (Savaş et al., 2008). Variations in measures used limits comparisons but help to illustrate the wide‐ranging long‐term impact associated with CRPS.

#### 3.3.5 Work status

De Mos et al in a population‐based study reported that of 102 patients, 53% had been working prior to CRPS onset. Of these 54 patients, only 41% of these had been able to return to their normal work, while 28% returned to work, requiring adaptation of work roles or hours and 31% stopped work altogether because of CRPS (Netherlands) (De Mos et al., 2009). Dumas et al used information from French occupational health clinics; reported 67% of 55 patients were able to RTW at one year, while 33% were unable due to CRPS. On univariate analysis, criteria predictive of RTW, a sedentary job, and high level of education (Dumas et al., 2011). Subbareo & Stillwell (USA) reported that for 125 patients, 61% of patients had been working prior to CRPS onset −31% of these retired or did not return to the same work due to CRPS, 35% were officially classed as disabled (although it is unclear how this affected their job), 30% returned to same jobs and 4% were not accounted for (Subbareo & Stillwell 1981). In Sharma et al’s study (USA) 62% of 888 patients reported that CRPS interfered with their work role. They also report that 2/3 of patients claimed benefits due to CRPS functional impairments (Sharma et al., 2009).

Results overall show roughly 1/3 of patients are unable to RTW at long‐term FU because of CRPS, while a further proportion of patients experience some work status compromise.

### 3.4 Cross sectional studies/correlation studies (*n* = 6)

Six identified studies used cross sectional sampling (Table 2) and considered the association of different variables with respect to CRPS outcome (Table 5) (Antunovich et al., 2020; Bean et al., 2014a; De Boer et al., 2011; De Jong et al., 2011; Schwartzman et al., 2009; Veldman et al., 1993), three had also been included within review®, (De Boer et al., 2011; Schwartzman et al., 2009; Veldman et al., 1993) none were excluded.

**TABLE 5:** Cross sectional and correlation study outcomes reported

Reference	n	Outcome timing *(Average duration since symptom onset)*	Outcome Domains				
			Recovery/severity	Sensory/Pain outcomes	Motor function outcomes	Physical and social disability outcomes	Work status outcomes
Bean et al. (2014a)	88	CRPS 43.25 months (±53.73) LBP 43.42 (±53.14)	n/a	CRPS group NRS = 7.59 (1.77) LBP group NRS = 7.69 (1.58) Pain intensity not correlated to pain duration	n/a	For both groups, pain intensity was predicted by kinesiophobia, and pain intensity and depression were predictive of disability	RTW = 22%
Antunovich et al. (2020)	53	62.14 months (±50.15)	n/a	NRS=6.42 (1.68) Pain intensity correlated to CRPS duration = β = 0.28; *p* = 0.039	n/a	Negative illness perceptions were associated with greater pain, disability, and kinesiophobia	18% employed. 43% on income compensation
De Jong et al. (2011)	Study 1 = 79 Study 2 = 109	Study 1< 1 month Study 2 >6 months	n/a	Study 1 NRS = 7.34 (1.73) Study 2 NPS = 50.16 (17.46)	n/a	Perceived harmfulness of activities significantly predicted functional limitations	n/a
Schwartzman et al. (2009)	656	1 to 46 years	n/a	NRS >5 years = 6.91 (0.5) NRS >15 years = 7.92 (0.6) Pain intensity correlated to CRPS duration = *r* = 0.60, *p* = 0.005	% of patients with loss of strength (sub) >5 years = 93% >10 years = 94% Strength was not correlated with duration = (*r* = 0.22, *p =* 0.340)	Reported problems with general activity, enjoyment of life, mood, work, ability to concentrate, and ability to sleep = 97%	81% of patients had stopped work at some time point 23% of these able to RTW
Veldman et al. (1993)	4 groups totalling 829	Group 1: 0–2 months (n = 156) Group 4: >12 months (n = 231)	n/a	% with pain Group 1‐<2months = 92% Group 4– 12 months = 97%	% of patients with reduced range of movement Group 1 = 90% Group 2 = 83%	n/a	n/a
De Boer et al. (2011)	4 groups totalling 692	Group 1: 0–2 months (*n* = 156) Group 4: >12 months (*n* = 231	n/a	% with pain Group 1‐<2months = 85% Group 4– 12 months = 95%	% of patients with reduced range of movement Group 1 = 77% Group 2 = 77% % of patients with reduced grip strength Group 2 = 67% (sub)	n/a	n/a

Note

All scores represent average scores for study population of stated measure.

Abbreviations: %, percentage of total sample; CRPS, complex regional pain syndrome; figures in brackets, standard deviation; n, number; n/a, no outcomes for this domain recorded; NRS, numerical rating scale; obj, objective measure; PI, pain intensity; RTW, return to work; sub, subjective measure.

#### 3.4.1 Sensory/pain outcomes

Antunovich et al found longer symptom duration predicted greater pain intensity (β = 0.28; *p* = 0.039, *n* = 53) (Antunovich et al., 2020). Schwartzman et al also demonstrated a positive correlation between pain intensity and disease duration (Schwartzman et al., 2009) (*r* = 0.60, *p *= 0.005, *n* = 656). In contrast Bean et al found disease duration was not predictive of pain or disability (Bean et al., 2014a).

Three studies reported high pain intensity and functional disability were positively correlated with patient’s perception of activity and movement (Antunovich et al., 2020; Bean et al., 2014a; De Jong et al., 2011). Bean et al and Antunovich et al reported higher pain intensity and disability were associated with higher kinesiophobia (TSK) scores (fear of pain due to movement) (Antunovich et al., 2020; Bean et al., 2014a), while De Jong et al reported these variables were associated with perceived harmfulness of activities (De Jong et al., 2011). Antunovich et al additionally found negative illness perceptions and a poorer understanding of CRPS were correlated with higher levels of pain intensity and disability (Antunovich et al., 2020).

#### 3.4.2 Motor function

Features of pain and motor dysfunction remained dominant and consistently high regardless of CRPS duration. For example, Veldman et al show limited movement was a feature for 90% at 2 months and 83% of cases >12 months (Veldman et al., 1993). De Boer et al using reported limited movement for 77% at both 2 and 12 months (IASP criteria). They also noted that 67% of patients had problems associated with reduced strength at 12 months (De Boer et al., 2011). Schwartzman et al found similarly found loss of strength remained a dominant feature of both early and longer duration CRPS. They reported 93% prevalence during the first 5 years and 94% prevalence after 10 years (Schwartzman et al., 2009). Interestingly despite the high prevalence loss of strength was not correlated with disease duration in this study (*r* = 0.22, *p *= 0.340).

#### 3.4.3 Physical and social disability

Three studies examined predictive variables associated with disability (see above).

Schwartzman reported that pain interfered with general activity, enjoyment of life, mood, work, ability to concentrate and ability to sleep in 97% of respondents (Schwartzman et al., 2009).

#### 3.4.4 Work status

Three studies included work related outcomes reporting higher proportions of people not being able to RTW than prospective and retrospective studies (Antunovich et al., 2020; Bean et al., 2014a; Schwartzman et al., 2009). Schwartzman et al reported that 81% of patients with an average CRPS duration of 37.5 years (*n* = 656, USA) had stopped work at some time point with only 27% returning to work in their normal capacity. Both New Zealand studies reported similar percentages of patients in work, Bean et al report 20% (*n* = 88, 43.15 months) (Bean et al., 2014a), Antunovich et al reports 18%, while 43% were received income compensation (*n* = 53, 62.4 months) (Antunovich et al., 2020). These proportions are lower than for other study types however it should be noted that these studies measured work status for populations who required ongoing longstanding CRPS treatment.

## 4 DISCUSSION

The aim of the review was to summarize the published data concerning the impact of CRPS symptoms, specifically the physical and occupational impact of symptoms, at 12 months from symptom onset and beyond. The 22 included studies agreed that features of CRPS usually improve with time. Pain and motor dysfunction were found to be the most prevalent ongoing symptoms affecting between 51%–89% of all patients at longer term follow up. The persistence of these features can dramatically impact a person’s physical and social abilities. How physical and social disability was measured was highly variable. Results indicate that CRPS is associated with a 25%–66% reduction in grip strength and prevents return to work for 30%–40% of cases of at ≥12 months. The current review provides first‐time quantitative data on function and work status for CRPS ≥12 months and builds on evidence provided by review® (Bean et al., 2014b).

### 4.1 Motor function

Across 14 studies, 51%–89% of patients continue experiencing symptoms of weakness, stiffness, and reduced range of movement at 12 months and beyond (Bean et al., 2016, Bickerstaff & Kanis, 1994, De Boer et al., 2011, De Mos et al., 2009, Ehrler et al., 1995, Galer et al., 2000, Geertzen et al., 1998, Laulan et al., 1997, Savaş et al., 2008, Schwartzman et al., 2009, Sharma et al., 2009, Subbarao & Stillwell, 1981, Veldman et al., 1993, Zyluk, 2001). This percentage range across studies is narrower than for any other symptom of CRPS. The consistently high prevalence of motor problems across all study types illustrates these are significant problems for people living with CRPS.

Recent studies were more likely to include objective measures of function (9/22), which allows us to report for the first‐time quantitative data relating to functional compromise. Prospective studies that included objective measures indicated that for all patients CRPS was associated with on average a 20%–25% (Bean et al., 2016; Laulan et al., 1997), reduction in range of movement and reduced grip strength of between 25%–50% at ≥12 months (Bickerstaff & Kanis, 1994; Laulan et al., 1997; Zyluk, 1998). Slightly higher reductions in grip strength at ≥12 months of up to 66% were reported by retrospective and cross‐sectional studies (De Boer et al., 2011; Ehrler et al., 1995; Geertzen et al., 1998, Savaş et al., 2008). This higher percentage is not unexpected given studies of this type are associated with higher confounding bias and selection bias than prospective studies. Regardless of study type these reductions in movement and grip strength, are not insignificant and will undoubtedly compromise a person’s ability to function. We speculate that the impact of such losses will have varying significance dependent on factors including occupation. For example, 50% loss of grip strength may make it harder for someone to return to a manual job than a desk job and have a greater impact for persons with a manual occupation. Future research should explore such factors to help identify and support patients at risk of a poorer outcome. These findings suggest an important focus of CRPS treatment should be the restoration of movement and grip strength. Research examining the efficacy of CRPS treatments, therefore, needs to include outcomes that measure a change in movement and grip strength and additionally consider the significance.

### 4.2 Work status (WS)

We were able to provide first‐time quantitative data on function and work status for CRPS ≥12 months. Seven studies measured WS in different countries with differing social systems of support (Bean et al., 2014a, Bean et al., 2016, De Mos et al., 2009, Dumas et al., 2011, Schwartzman et al., 2009, Sharma et al., 2009, Subbarao & Stillwell, 1981). Despite these differences, the results across these studies were consistent indicating that a percentage range of 60%–70% of patients working prior to onset of CRPS return to work and 30%–40% do not based on binary outcomes. Notably, the proportion that does not RTW is about double that of patients classed as ‘persistent’ CRPS, that is, patients that do not get better (Goebel et al. 2018), suggesting that despite experiencing some improvements a sizable proportion of patients will not RTW at all. For those patients who return to work there is some suggestion that a proportion of these struggle, with a small number of retrospective studies suggesting that 27%–35% of patients who did RTW required work role adaptations (De Mos et al., 2009; Subbarao & Stillwell, 1981). A further study reported at FU that patients were working on average only 25.97 h per week (although it is unclear whether this represented reducing working hours) (Bean et al., 2016). Therefore, some studies provide the suggestion that CRPS further comprises work status however the quality of data was often limited. Future studies should attempt to measure any required changes to maintain working status including the impact on working hours. Overall, studies would suggest that between 30%–40% of working patients will not RTW and a further 27%–35% would require some form of workplace adaptation after a period of CRPS. This number is comparably higher than the 11% reported after an episode of chronic low back pain (Costa et al., 2009). Results, therefore, suggest the current health economic impact in terms of occupational recovery is not yet fully understood. What factors influence return to or the need for adaptation of work, and whether there are any health inequalities relating to the types of work remains unclear; further work is needed to understand this.

### 4.3 Physical and social disability

There was a great deal of heterogeneity in outcomes used to measure general disability. For example, retrospective studies reported that between 62%–86% of patients had interference with multiple and varied aspects of ADLS far beyond 12 months. The wide range of different outcome measures illustrates the wide‐ranging impact of CRPS but also represents a significant limiting factor of the reviewed literature. The need for researchers to reach a consensus on outcome measures has been a debated topic for some time with the Core Outcome Measurement set for complex regional PAin syndrome Clinical sTudies (COMPACT) representing work to tackle this issue (Grieve et al., 2017).

### 4.4 Prognostic indicators related to outcome

In concordance with review® high pain intensity was frequently shown as a prognostic marker of poor outcome. Since review® a further correlation study has been published that considered prognostic indicators of recovery (Bean et al., 2014a). Together the correlation studies identify psychological variables of fear of pain with movement, negative illness perception and a poorer understanding of CRPS to be positively correlated with outcomes of pain and disability. This thereby might provide some direction in terms of treatment targets, however, alternatively, these variables might be biologically intrinsically linked to the respective subtypes providing no obvious handle for improvement.

### 4.5 In the context of review®

Although this review includes many of the same references as review® by Bean et al, some (*n* = 3) were excluded due to a slightly more stringent inclusion criterion. Furthermore, studies were evaluated using a different quality assessment tool and therefore interpreted somewhat differently. Results from this review concur with review® 10 years ago indicating that CRPS often improves in the initial 12 months and motor dysfunction are the most persistent disabling features of CRPS. Since review® there were some clear improvements in study qualities. For example, we highlight a greater proportion of studies reporting statistical differences when measuring changes 11/22 and used validated outcome measures 12/22.

### 4.6 Limitations

As with review®, we identified several limitations in the literature such as high attrition rates and low response rates across retrospective studies are a particular source of bias. This limits the generalizability of findings of included retrospective studies, and study attrition should be considered within future reviews.

Diagnostic criteria between studies were also not uniform. Differing criteria reflect different inclusivity and exclusivity and contribute to variable levels of selection bias and the again the generalizability of findings. The Budapest criteria became the recognized gold standard diagnostic criteria in 2012, and 5/8 studies published after this date used these criteria. Future reviews may wish to include only studies published after this date.

We found that most studies (14/22) did not measure CRPS from onset (inception cohorts), therefore, information relating to the natural course of CRPS within the review is limited.

A limitation of this review was that only one reviewer searched the literature with a second person checking data extraction and quality assessment which therefore increases potential interpretation bias. The inclusion of a further reviewer would mitigate this in future reviews.

### 4.7 Conclusion

Results from this review concur with review® 10 years ago indicating pain and motor dysfunction are the most persistent disabling features of ongoing CRPS. We now also report first‐time quantitative data specific evidence about losses to motor function, long‐term compromises to work status. Results indicate despite general improvements in features of CRPS, the ongoing impact of CRPS on hand function and work status is relatively high. Future research should explore what drives limitation to function and work status and if and how these limitations can be prevented.

## CONFLICTS OF INTEREST

All authors wish to declare no conflicts of interest.

## AUTHORS CONTRIBUTIONS

SJ, AG, planned and conducted the literature review, SJ prepared the manuscript with AG, FC and SG providing further editing. All authors have discussed the results and commented on the manuscript.

## Supporting information

Supplementary Material
