# Supplementary material for: Complex regional pain syndrome what is the outcome? ‐ a systematic review of the course and impact of CRPS at 12 months from symptom onset and beyond
Source: Eur J Pain. 2022 May 4;26(6):1203–20. doi: 10.1002/ejp.1953 (PMC9324966; doi:10.1002/ejp.1953)
Supplement: Supplementary file 1 — Supplementary Material [file EJP-26-1203-s001.docx]

Supplementary materials:

Supplement 1: Example Search strategy

Supplement 2: Table S1: Inclusion criteria screening tool

Supplement 3: Table S2: Joanna Briggs institute (JBI) CRITICAL APPRAISAL CHECKLIST FOR STUDIES REPORTING PREVALENCE DATA.

Supplement 4: Table S3: Results of studies measuring sudomotor and vasomotor outcomes.

Supplement 1:

Example Search strategy:

EMBASE

1. *"COMPLEX REGIONAL PAIN SYNDROME"/ OR *"COMPLEX REGIONAL PAIN SYNDROME TYPE I"/ OR *"COMPLEX REGIONAL PAIN SYNDROME TYPE II"/ Peripheral neuropathic pain

2. *"COMPLEX REGIONAL PAIN SYNDROME TYPE I"/ OR *ALGODYSTROPHY/

3. exp EPIDEMIOLOGY

4. *INCIDENCE/

5. *"FOLLOW UP"/ OR *"EVALUATION AND FOLLOW UP"/

6. (prognos*).ti,ab

7. (predict*).ti,ab

8. (course).ti,ab

9. Outcome*

10. 1 OR 2

11. 3 OR 4 OR 5 OR 6 OR 7 OR 8 OR 9

12. 10 AND 11

13. Date limit 2012-2021

14. Limit (English language and humans, adults)

Supplement 2:

| Table S1: Inclusion criteria screening tool | | |
| --- | --- | --- |
|  | **Include** | **Exclude** |
| Population | - Adults CRPS/ Sudecks/RSD - 12 months - DRF | - Pediatric - not CRPS/sudecks/RSD - <12-month FU |
| Intervention | - Outcome, - incidence, - natural history, - severity, - course, - predictive factors, - prognosis | - Treatment intervention |
| Comparator | - None required | - n/a |
| Outcomes | Regarding signs and symptoms   - Diagnosis at different time points - Symptom severity - Symptom resolution - Symptom duration   Regarding Recovery   - self-report/ - health (physical and mental health, health related quality of life) - pain (e.g., pain intensity, etc.), - physical functioning (e.g., disability, limitations of activities of daily living (ADL), level of physical activity, etc.), - social disability (e.g., changed social role, work disability, sick leave, disability pension, return to work, care-seeking, medication, etc.). |  |
| Study design | - all | - Analyzed sample size <20 - Response/FU rates <50% |
| Language | - English | - Non-English |
| Other limits |  | - Abstract only |
| Notes |  | |

Supplement 3:

| Authors | Was the sample frame appropriate to address the target population? | Were study participants sampled in an appropriate way?  Sampling method described | Inception cohort | Was the sample size adequate? | Were the study subjects and the setting described in detail? | Was the data analysis conducted with sufficient coverage of the identified sample? | Were valid methods used for the identification of the condition?  Diagnostic criteria recognized | Was the condition measured in a standard, reliable way for all participants?  a) Outcome defined?  b) Outcome objective  c) standardized validated? | Was there appropriate statistical analysis?  Level of significance? | Was the response rate adequate, and if not, was the low response rate managed appropriately?  Attrition <20%, response rate >75% |
| --- | --- | --- | --- | --- | --- | --- | --- | --- | --- | --- |
| Prospective |  |  |  |  |  |  |  |  |  |  |
| Beerthuizen^8^ | N, Fracture | Y | Y | U-timeframe | Y | Y | Y- 3 diagnostic criteria | a) Y  b) Y  c) Y | Y | Y 17% |
| Bickerstaff^10^ | N, one type of fracture | Y | Y | Y | Y | Y | Y | a) Y  b) Y  c) Y | Y | U |
| Laulan^27^ | N, one type of fracture | Y | Y | U-? 7 months | Y | Y | N | a) Y  b) Y  c)N | N | Y 18% |
| Bean^6^ | N, pain clinic | Y | N | U | Y | Y | Y | a) Y  b) Y  c) Y | Y | Y 5% |
| Bean^7^ | N, pain clinic | Y | N | U | Y | Y | Y | a) Y  b) N  c) Y | Y | Y 5% |
| Zyluk^39^ | Y | Y | Y | U | Y | Y | Y | a) Y  b) Y  c) Y | N | Y 10% |
| Retrospective |  |  |  |  |  |  |  |  |  |  |
| Gougeon^23^ | N | N | U | N | N | U | N | a) Y  b) N  c)N | N | N- 61% |
| Bejia^9^ | N | Y | N | N | N | U | N | a) Y  b) N  c)N | N | U |
| De Mos^15^ | Y | Y | Y | Y | Y | Y | Y | a) Y  b) N  c)N | Y | N- 62% |
| Dumas^16^ | Y | Y | N | Y | N- limited descript demographics- | U | Y | a) Y  b) N  c)N | N | U |
| Geertzen^20^ | N, rehab clinic | Y | N | Y | Y | Y | N | a) Y  b) Y  c)N | Y | N-70% |
| Subbarao^36^ | N, pain clinic | Y | N | Y | Y | U | N | a) Y  b) N  c)N | N | N- 63% |
| Galer^19^ | N, pain clinic | Y | N | N- due to low response rate | Y | N- due to low response rate | Y | a) Y  b) N  c)Y | N | N-56% |
| Sharma^35^ | N, online support group | Y | N | N | Y | U | Y | a) Y  b) N  c)N | N | N 1359/6000 active users |
| Savas^33^ | N, rehab clinic | Y | N | U | Y | Y | Y | a) Y  b) Y  c)N | Y | Y |
| Ehrler^17^ | N | N | N | U | N | N | U | a) N  b) Y  c)N | N | N- 53% responded |
| Cross sectional & Correlation |  |  |  |  |  |  |  |  |  |  |
| Bean^4^ | N, pain clinic | Y | N | Y | Y | Y | Y | a) Y  b) N  c)Y | Y | N-but appropriately managed as reran tests with 60 in each group with no change to affect. |
| Antunovich^2^ | N, pain clinic | Y | N | Y | Y | Y | Y | a) Y  b) N  c)Y | Y | Y |
| De Jong^14^ | N, pain clinic | Y | N | N | Y | N- due to low response rate | Y | a) Y  b) N  c)Y | Y | N-27% of entire sample responded |
| Schwartzman^34^ | N, Chronic | Y | N | Y | Y | Y | Y | a) Y  b) N  c)Y | Y | Y |
| Veldman^38^ | Y | Y | N | Y | Y | Y | Y | a) Y  b) N  c)Y | N | Y |
| De Boer^13^ | Y | Y | N | Y | Y | U-as response rate unclear | Y | a) Y  b) Y  c)Y | Y | U |
| Table S2: Joanna Briggs institute (JBI) CRITICAL APPRAISAL CHECKLIST FOR STUDIES REPORTING PREVALENCE DATA. Possible outcomes Y=yes, N= No, U= unclear | | | | | | | | | | |

Supplement 4:

| Reference | n |  | Measure | Timepoints | Result |  |  |
| --- | --- | --- | --- | --- | --- | --- | --- |
| **Prospective studies** | | | |  | T1 | T2 | T3 |
| Beerthuizen^8^ | 596 |  | n/a |  | n/a | n/a | n/a |
| Bickerstaff^10^ | 77 | V | % Reporting features | T1-3 months  T2 6 months  T3 12 months | 91% | 65% | 29% |
|  |  | S | % Reporting features |  | 87% | 29% | 12% |
| Bean^6^ | 66 | V | % Reporting colour asymmetry | T1- <12 weeks  T2 6 months  T3 12 months | 97% | 65% | 52% |
|  |  | V | % Reporting temperature asymmetry |  | 62% | 72% | 59% |
|  |  | S | % Reporting sweating asymmetry |  | 68% | 45% | 35% |
|  |  | S | % Reporting swelling asymmetry |  | 98% | 67% | 48% |
| Laulan^27^ | 26 | V&S | Mean values of sympathetic parameter of the clinical score for each CRPS subgroup  (scored 2-5- where 2 denotes no features) | T1- < 1weeks  T2- 12 months | Active 3.2  Transient 4.9  Borderline 2.4 |  | Active 4.8  Transient 2.3  Borderline 2.1 |
| Bean^7^ | 59 | V | % with colour asymmetry  % with temperature asymmetry | T1-< 12 weeks  T2- 6 months  T3- 12 months | 96%  62% | 66%  72% | 52%  58% |
|  |  | S | % with Swelling asymmetry  % with sweating asymmetry |  | 96%  68% | 68%  45% | 48%  35% |
| Zyluk^39^ | 30 | V | Number of patients with discoloration  Number of patients temperature changes | T1-< 12 weeks  T2- 6 months  T3- 13 months | 22/30  27/30 | 13/28  15/28 | 5/27  7/27 |
|  |  | S | Number of patients swelling |  | 26/30 | 6/28 | 4/27 |
| **Retrospective studies** | | | | Mean Follow up  Time points | Baseline | FU |  |
| Dumas^16^ | 55 | V | Correlation of vasomotor symptoms in respect to return to work | 12 months | - |  | No correlation |
|  |  | S | Correlation of symptoms of swelling in respect to return to work |  | - |  | P=0.01 |
| Galer^19^ | 55 | S | % Reporting change over time in swelling | 3.3 years | - | Improved= 51.6%  Unchanged=38.7%  Worse=9.7% |  |
| De Mos^15^ | 102 | V | % Reporting features | 5.8 years | - | 22% |  |
|  |  | S | % Reporting features |  |  | 19% |  |
| Gougeon^23^ | 573 |  | n/a |  |  |  |  |
| Bejia^9^ | 60 |  | n/a |  |  |  |  |
| Geertzen^20^ | 65 |  | n/a |  |  |  |  |
| Subbarao^36^ | 125 |  | n/a |  |  |  |  |
| Sharma^35^ | 888 | V | % Decrease in temperature disturbance from onset to time of survey  % Decrease in colour disturbance from onset to time of survey | 5.5 years | - | 9.3%  18.2% |  |
|  |  | S | % Decrease in swelling disturbance from onset to time of survey |  |  | 20% |  |
| Savas^33^ | 30 |  | Number of patients with abnormal sweating at FU | 1.5 years | - | 5 |  |
| Ehrler^17^ | 31 |  | n/a |  |  |  |  |
| **Cross sectional & correlation studies** | | | |  |  |  |  |
| Veldman^38^ | 231 | S | % Reporting features of swelling | T1 < 2months  T2 12 months | 86% | 55% |  |
| De Boer^13^ | 352 | S | % Reporting features of swelling |  | 60% | 48.7% |  |
| Schwartzama^34^(2) | 656 | V | Correlation between colour changes & disease duration |  | p=0.004 |  |  |
|  |  | V | Correlation between temperature changes & disease duration |  | p=0.05 |  |  |
|  |  | S | Correlation between swelling changes & disease duration |  | p=0.50 |  |  |
| Bean^4^ | 88 |  | n/a |  |  |  |  |
| Antunovich^2^ | 53 |  | n/a |  |  |  |  |
| De Jong^14^ | Study 1=79  Study 2=109 |  | n/a |  |  |  |  |
| Table S3: Results of studies measuring sudomotor and vasomotor outcomes. | | | | | | | |
